# A rare case of pseudoaneurysmal hemorrhage, necrotizing fasciitis, and costochondritis after pancreaticoduodenectomy

**DOI:** 10.1186/s40792-022-01418-5

**Published:** 2022-04-24

**Authors:** Lulu Li, Kyohei Abe, Tomoyoshi Okamoto, Michinori Matsumoto, Yasuro Futagawa, Masaru Kanehira, Toru Ikegami

**Affiliations:** 1 Department of Surgery, The Jikei University School of Medicine, 3-25-8 Nishishimbashi, Minato-ku, Tokyo, Japan; 2 Department of Surgery, The Jikei University Daisan Hospital, 4-11-1 Izumihoncho, Komae, Tokyo Japan; 3 Division of Hepatobiliary and Pancreatic Surgery, Department of Surgery, The Jikei University School of Medicine, 3-25-8 Nishishimbashi, Minato-ku, Tokyo, Japan

**Keywords:** Cholangiocarcinoma, Pancreaticoduodenectomy, Necrotizing fasciitis, Chemotherapy

## Abstract

**Background:**

Necrotizing fasciitis after pancreaticoduodenectomy (PD) has never been reported. We experienced a case of necrotizing fasciitis caused by pseudoaneurysmal hemorrhage after PD.

**Case presentation:**

A 72-year-old male was diagnosed with cholangiocarcinoma and underwent PD. Bile leakage was detected postoperatively, conservatively resolved, and the patient was discharged on the 36th day after surgery. On the 42nd day after surgery, a pseudoaneurysm of the gastroduodenal artery ruptured. Transcatheter arterial embolization was performed for hemostasis: however, a large intra-abdominal abscess caused by an infected hematoma was recognized. On the 57th day after surgery, the patient developed necrotizing fasciitis. He underwent debridement with skin reconstruction using a latissimus dorsi flap and skin transplantation. Costochondritis and liver metastasis were detected on the 267th day after surgery. Infection was controlled by rib cartilage resection, debridement, and negative pressure wound therapy. Chemotherapy involving gemcitabine and cisplatin was initiated on the 460th day after the initial surgery with a partial response (PR) and was continued for more than one year.

**Conclusions:**

We herein reported a rare case of necrotizing fasciitis following hematoma infection after PD that was treated using multidisciplinary therapy with PR following chemotherapy.

## Background

Postoperative hemorrhage is one of critical complications that may occur after pancreaticoduodenectomy (PD). More than 90% of cases occur within 5 weeks of surgery [1]. There is currently no information on necrotizing fasciitis after PD. We herein report a case of necrotizing fasciitis caused by pseudoaneurysmal hemorrhage on the 6th week after PD. A partial response (PR) was achieved and maintained with chemotherapy after multidisciplinary therapy.

## Case presentation

A 72-year-old male with right costalgia and fever presented to the department of gastroenterology and hepatology in our hospital. His previous medical history was unremarkable. After relevant examinations, extrahepatic cholangiocarcinoma was suspected.

**Fig. 1:**
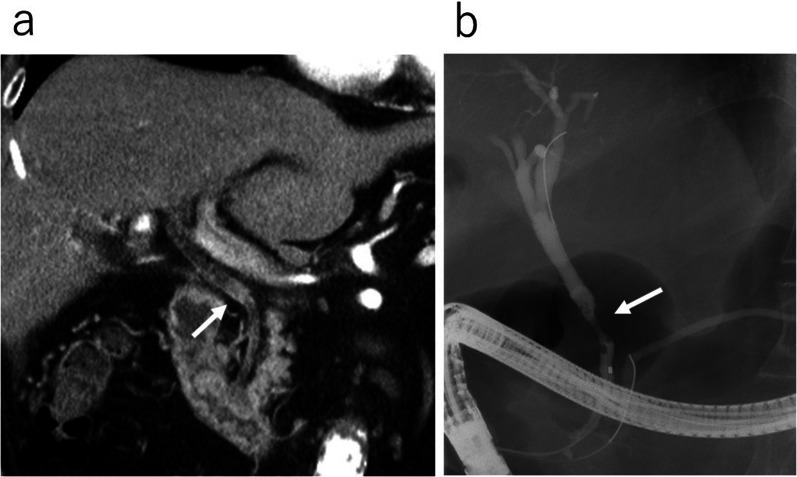
Image findings of distal bile duct cancer. **a** Contrast-enhanced CT before PD and after percutaneous transhepatic gallbladder drainage (PTGBD) showing a 2-cm-long mass with contrast enhancement in the central bile duct (arrow →). **b** ERCP before PD showing the narrowing of the distal bile duct (arrow ←) without the visualization of the cystic duct. The cytology of the bile duct brush and bile cytology were both class III

Contrast-enhanced computed tomography (CT) showed a 2-cm-long mass with contrast enhancement in the central bile duct. There were no findings suggestive of enlarged lymph nodes or distant metastasis (Fig. 1a). Endoscopic retrograde cholangiopancreatography (ERCP) findings revealed narrowing of the central bile duct, without the cystic duct being visualized. The cytology of the bile duct brush and bile cytology were both class III (Fig. 1b).

The patient was diagnosed with distal bile duct cancer classified as cT2N0M0, Stage IB [2]. On the 21st day after the first visit, he was referred to the department of surgery. Blood biochemical tests at this time showed mild liver dysfunction. The serum level of carbohydrate antigen 19-9 was 55 IU/mL, which was slightly elevated. The patient underwent subtotal stomach-preserving PD on the 46th day after the first visit. The duration of surgery was 7 h and 52 min with a blood loss volume of 430 mL. 7.5Fr tube was inserted as the pancreatic duct stent for external drainage across the jejunum and removed 3 weeks after the surgery. We also inserted 3 mm internal stent as the biliary stent. Reconstruction following PD was performed by the modified Child method. Pancreaticojejunostomy was performed by the pancreaticojejunal mucosal anastomosis and the modified Kakita method. Choledochojejunostomy was performed with 24 stitches of 4-0 polydioxanone suture. 10 mm soft drains were inserted into the back side of the choledochojejunostomosis and the pancreaticojejunostomosis, respectively. Histopathological findings revealed well-differentiated tubular adenocarcinoma with a flat invasive lesion with indistinct borders in the distal bile duct, which was classified as fT2N0M0, Stage IB [2]. Neuroendocrine carcinoma was detected in some parts of the lesion, which indicated mixed adenoneuroendocrine carcinoma. The hepatic margin of the bile duct was negative.

Drainage amylase level of the pancreaticojejunal drain was 336 U/L and drainage total bilirubin level at the same site was 0.4 mg/dL, respectively, on the 3rd day after surgery. Drainage amylase level of the choledochojejunal drain was 42 U/L and drainage total bilirubin level at the same site was 39 mg/dL, respectively, on the same day. The serum bilirubin level at the same day was 1.0 mg/dL. Therefore, the patient was diagnosed with bile leakage [3].

A liquid diet was given on the 4th day after surgery. Drainage amylase level of the pancreaticojejunal drain was 4 U/L and drainage total bilirubin level at the same site was 21.8 mg/dL, respectively, on the 5th day after surgery. Drainage amylase level of the choledochojejunal drain was 163 U/L and drainage total bilirubin level at the same site was 1.2 mg/dL, respectively, on the same day. The serum bilirubin level at the same day was 0.8 mg/dL.

We removed the pancreaticojejunal drain and replaced the choledochojejunal drain on the 7th day after surgery. Drainage amylase level of the choledochojejunal drain was 14 U/L and drainage total bilirubin level at the same site was 7.1 mg/dL, respectively, on the same day. The serum bilirubin level at the same time was 0.6 mg/dL. As he was asymptomatic and the results of blood test on the 7th day after surgery were within normal limits as postoperative, we started to gradually increase the amount of solid diet. He ate almost half the amount.

Drainage amylase level of the choledochojejunal drain was 662 U/L and drainage total bilirubin level was 35.7 mg/dL on the 14th day after surgery. Therefore, the patient was diagnosed with pancreatic fistula [4]. The patient recovered conservatively with drain replacement and antimicrobial therapy, and he was discharged on the 36th day after surgery.

**Fig. 2:**
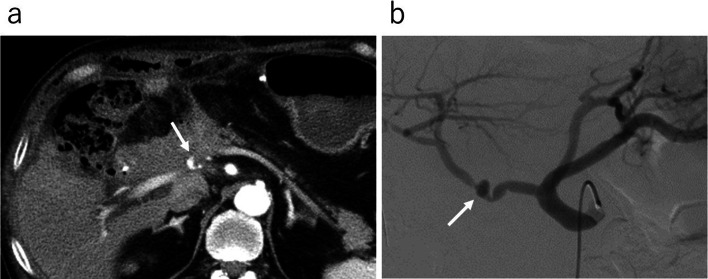
Image findings of pseudoaneurysm after PD. **a** Contrast-enhanced CT showing the formation of a pseudoaneurysm at the end of the GDA (arrow →). There was widespread hematoma in the surrounding area. **b** Angiography showing a pseudoaneurysm at the end of the GDA (arrow →)

**Fig. 3:**
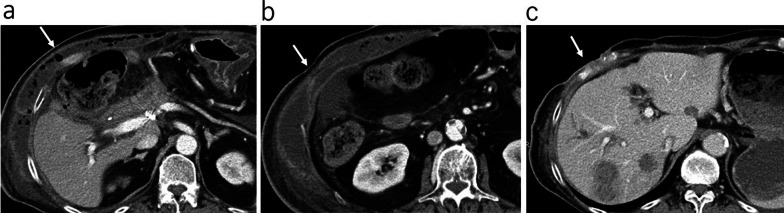
CT findings. **a** Contrast-enhanced CT showing necrotizing fasciitis and costochondritis of the right oblique abdominal muscle (arrow →). **b** Contrast-enhanced CT showing that the infected hematoma contacted to inner abdominal wall (arrow →). **c** Contrast-enhanced CT showing multiple liver metastases of 12 to 16 mm in diameter in both lobes. A subcutaneous abscess was detected on the right 11th rib (arrow →)

**Fig. 4:**
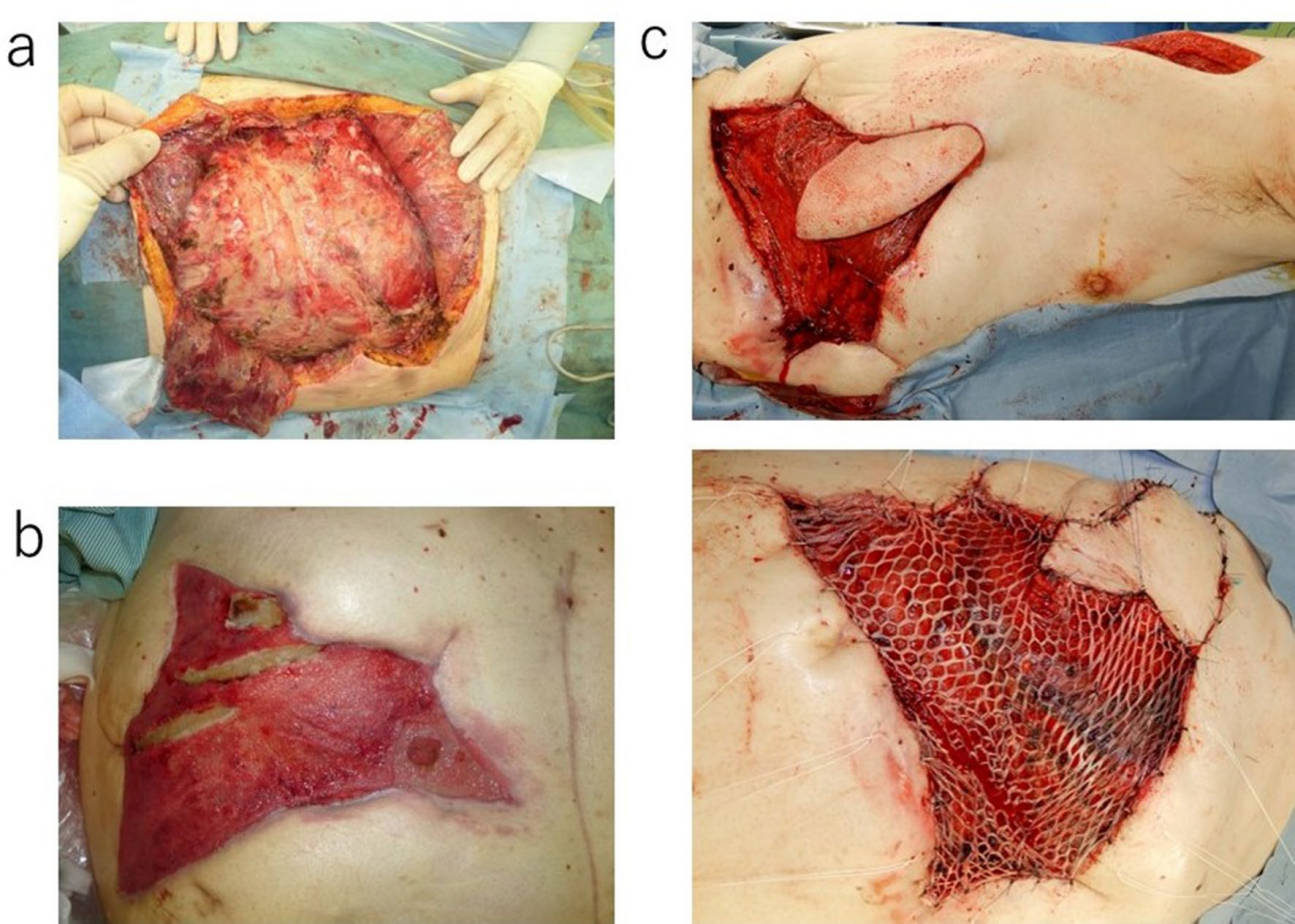
**a** The infected area was incised and opened on the 16th day after TAE. **b** NPWT was started on the 16th day after TAE. **c** The patient underwent debridement of the infected area on the 78th day after TAE, and simultaneous flap plasty with musculus latissimus dorsi (above) and skin grafting from the right buttock were performed (below) to close the wound from the right anterior chest to the upper abdomen in collaboration with the department of plastic surgery

**Fig. 5:**
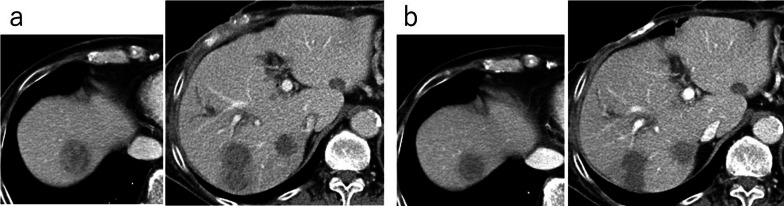
Contrast-enhanced CT showing the therapeutic effect of PR after six courses of GC. **a** Before chemotherapy. **b** After six courses of GC

On the 44th day after surgery, abdominal pain developed in the right upper quadrant and the patient visited our outpatient clinic. Blood tests showed a white blood cell count of 16,400/μL, a hemoglobin level of 7.1 g/dL and a C-reactive protein level of 13.24 mg/dL. Contrast-enhanced CT revealed a pseudoaneurysm at the end of the gastroduodenal artery (GDA) with widespread hematoma in the surrounding area (Fig. 2a). Angiography also showed the pseudoaneurysm at the end of the GDA (Fig. 2b). Transcatheter arterial embolization (TAE) was performed through the GDA with a stretch-resistant coil for hemostasis. Although the patient did not rebleed after TAE, a large intra-abdominal abscess formed mainly in the right upper abdomen due to an infected hematoma. The patient developed necrotizing fasciitis of the right oblique abdominal muscle and costochondritis on the 13th day after TAE (Fig. 3a). The CT on the same day showed that the infected hematoma contacted to inner abdominal wall (Fig. 3b). Treatment with incisional drainage was started immediately with two subcutaneous 10 Fr Penrose drains. MRSA and Enterobacter species were detected in the wound on the 15th day after TAE and antimicrobial agents (vancomycin and clindamycin) were administered. The serum albumin level was 1.6 g/dL and extreme nutritional disturbance was observed. On the next day, we started to treat with debridement and negative pressure wound therapy (NPWT) (Fig. 4a, b). However, it was difficult to control the infection. The patient underwent debridement of the infected area on the 78th day after TAE, and simultaneous flap plasty with musculus latissimus dorsi and skin grafting from the right buttock were performed to close the wound from the right anterior chest to the upper abdomen in collaboration with the department of plastic surgery (Fig. 4c). The culture of the wound turned negative on the 98th day after TAE. Following postoperative wound care and rehabilitation, the patient was discharged on the 192nd day after TAE and followed up in the outpatient clinic. A small amount of discharge was observed from the wound on the 215th day after surgery and antimicrobial agent (levofloxacin) was administered. The discharge disappeared 3 weeks later. Multiple liver metastases were detected by CT on the 267th day after PD. Although chemotherapy was considered, CT also showed a subcutaneous abscess in the right 11th rib (Fig. 3c). Rib cartilage resection and debridement for right costochondral osteomyelitis were performed to control the infection. NPWT was postoperatively continued and resolved wound infection. Since rehabilitation and nutritional therapy were also performed during this period, the activities of daily living improved (Performance status 0). Therefore, chemotherapy with gemcitabine with cisplatin (GC) was initiated on the 460th day after PD. The patient received six courses of GC without serious adverse events. The therapeutic effect was assessed as PR (Fig. 5).

## Discussion

Pseudoaneurysmal rupture is a relatively specific complication after PD and has been reported to occur in 1.8–4.6% of patients [5]. Pseudoaneurysms often form at the end of the GDA or hepatic arteries that have been weakened by pancreatic fistulas or abscess formation [6]. In the present case, a pseudoaneurysm formed in the GDA, which appeared to be due to bile leakage after PD, resulting in intra-abdominal hemorrhage. Based on the clinical course the rupture of the pseudoaneurysm resulted in extensive intra-abdominal hematoma reaching inner abdominal wall (Fig. 3b), which developed into intra-abdominal and subcutaneous abscesses. Subcutaneous emphysema was already present in the CT imaging on the 44th day after surgery, suggesting the intra-abdominal infection caused by the bile leakage had probably already existed. Thus, secondary hematoma infection may have caused. Insufficient drainage of the infected hematoma may have allowed it to develop into necrotizing fasciitis. In addition, low nutritional status and cancer-bearing state were thought to have affected delayed wound healing after debridement. In the Clavien–Dindo classification, pancreatic fistula and bile leakage were diagnosed as IIIa and postoperative bleeding as IIIa in this case.

In this case, costochondritis was also developed following necrotizing fasciitis. The wound of debridement of necrotizing fasciitis and the rib that showed signs of infection were contiguous, suggesting that necrotizing fasciitis had developed into costochondritis. The fact that MRSA was again detected in the wound suggests that the previous infection may not have completely resolved. Despite wound care, shower cleansing, and appropriate antimicrobial therapy, the patient was considered to have been in a carcinomatous state and might be easily infected.

Previous findings on postoperative pseudoaneurysmal hemorrhage in the hepatobiliary and pancreatic regions indicated that hemorrhage occurred within 2 weeks of surgery in most cases [7], with more than 90% of cases of postoperative hemorrhage after PD occurring within 5 weeks of surgery [1]. Delayed hemorrhage occurred 6 weeks after surgery in the present case, which is relatively rare. Bleeding due to the rupture of a pseudoaneurysm after PD is fatal with mortality rates ranging between 7.4 and 22.2% [5]. Urgent TAE was effective for hemostasis in the present case, which was performed within approximately 18 h of the onset of symptoms.

The incidence of intra-abdominal abscess associated with PD was previously shown to be between 1.2 and 14%. Aranha et al. reported that the incidence of intra-abdominal abscess in cases without pancreatic fistulas was 1.8%, while it was 31% in cases with pancreatic fistulas [8].

Necrotizing fasciitis is characterized by necrosis of the superficial fascia and surrounding tissues, which rapidly progresses and may lead to serious systemic diseases, such as sepsis and multiple organ failure. Trauma, hemorrhoidal fistula, laparotomy, and intramuscular injection are common causes of necrotizing fasciitis. Surgical treatments, including debridement and drainage of the necrotic area, need to be performed as soon as possible in addition to the administration of antimicrobials [9]. However, limited information is currently available on postoperative necrotizing fasciitis [10–12]. A Pubmed search for “pancreaticoduodenectomy” and “necrotizing fasciitis” yielded zero relevant articles, which means that this case may be helpful for the future treatment of postoperative intra-abdominal abscesses after PD. There have been some case reports of necrotizing fasciitis after colorectal surgery. Miura et al. suggested the cause of necrotizing fasciitis after laparoscopic high anterior resection to be an infected hematoma or the development of emphysema during drain insertion [9]. Although postoperative necrotizing fasciitis is rare, it may occur due to an infected hematoma, similar to the present case. Early drainage, antimicrobial therapy, and surgical interventions need to be considered depending on the treatment course.

In this case, once we confirmed that necrotizing fasciitis and costochondritis, which developed secondary to PD, were under control, GC therapy was administered for recurrence of cholangiocarcinoma. There are reports that immunocompromised patients might be more prone to cancer recurrence [13–15] and there is a possibility that immunodepression due to postoperative complications may be involved in the recurrence of cholangiocarcinoma in this case. Difficulties were associated with decision-making regarding the initiation of chemotherapy for recurrent cancer due to complications. However, chemotherapy was initiated in the present case after successful multidisciplinary therapy. The patient continued to receive chemotherapy for more than 1.5 years and had a good quality of life after treatment completion.

## Conclusions

We herein reported a rare case of necrotizing fasciitis following hematoma infection after PD that was treated with multidisciplinary therapy with PR following chemotherapy. We also reviewed the relevant literature.

## Data Availability

The data supporting the conclusions of this article are included within the article.

## Abbreviations

PD: Pancreaticoduodenectomy
PR: Partial response
CT: Computed tomography
ERCP: Endoscopic retrograde cholangiopancreatography
GDA: Gastroduodenal artery
TAE: Transcatheter arterial embolization
NPWT: Negative pressure wound therapy
GC: Gemcitabine with cisplatin
PTGBD: Percutaneous transhepatic gallbladder drainage
